# Human thinking, shared intentionality, and egocentric biases

**DOI:** 10.1007/s10539-015-9512-0

**Published:** 2015-12-01

**Authors:** Uwe Peters

**Affiliations:** King’s College London, London, UK

**Keywords:** Human thinking, Shared intentionality, Explicit versus implicit, Egocentric bias

## Abstract

The paper briefly summarises and critiques Tomasello’s (2014) *A Natural History of Human Thinking*. After offering an overview of the book, the paper focusses on one particular part of Tomasello’s proposal on the evolution of uniquely human thinking and raises two points of criticism against it. One of them concerns his notion of thinking. The other pertains to empirical findings on egocentric biases in communication.

There is evidence that a number of non-human animals, ranging from corvids, domestic pigs, and dolphins to great apes, are capable of high-level thinking that is in many ways familiar from that in our own species (see, e.g. Taylor 2014; Marino and Colvin 2015; Herzing and Johnson 2015; Osvath and Martin-Ordas 2014). If that is so, what makes human thinking unique and what explains its origin?

In his recent book *A Natural History of Human Thinking*, Michael Tomasello sets out to offer answers to these questions. In what follows, I briefly summarise and critique the book.

I begin by clarifying what Tomasello means by ‘human thinking’ (“The notion of human thinking” section), before outlining the overall argument of the book (“Overview of *A Natural History of Human Thinking*” section). After that, I hone in on one particular part of Tomasello’s proposal on the evolution of uniquely human thinking and raise two points of criticism against it (“Critical discussion” section). One of them concerns his notion of thinking. The other pertains to empirical findings on egocentric biases in communication.

## The notion of human thinking

In *A Natural History of Human Thinking*, Tomasello’s goal is to offer an account of the unique nature and origin of human thinking. To specify what he means by ‘thinking’, Tomasello appeals to dual-process theory. He writes that although

humans and other animals solve many problems and make many decisions based on evolved intuitive heuristics (so-called system 1 processes), humans and at least some other animals also solve some problems and make some decisions by thinking (system 2 processes; e.g. Kahneman 2011). (2014: 4)

In Kahneman’s (2011) dual-process account, which Tomasello here endorses, system 1 processes are *inter alia* automatic and unconscious, i.e. working-memory independent processes, whereas system 2 processes are *inter alia* subject-controlled and conscious, i.e. working-memory dependent in nature (see Kahneman 2011: 18, 22, 25, 308). Given this, for Tomasello, thinking is a subject-controlled, conscious process.

More specifically, he holds that thinking is a single such process with three key components: “(1) the ability to cognitively represent experiences to oneself ‘off-line’; (2) the ability to simulate or make inferences transforming these representations causally, intentionally and/or logically; and (3) the ability to self-monitor and evaluate how these simulated experiences might lead to specific behavioural outcomes” (2014: 4).

Turning from thinking in general to human thinking, in particular, Tomasello holds that with respect to (1) to (3), unlike other animals, “only humans” are able to (i) cognitively represent and conceptualise identical situations or entities under “differing, possibly conflicting social perspectives (leading ultimately to a notion of ‘objectivity’)”, (ii) “make socially recursive and self-reflective inferences about others’ or their own intentional states”, and (iii) “self-monitor and evaluate their own thinking with respect to the normative perspectives and standards (‘reasons’) of others or the group” (ibid).

Tomasello calls the uniquely human thinking characterised by (i)-(iii) “objective-reflective-normative thinking” (ibid). His aim in *A Natural History of Human Thinking* is to offer an evolutionary explanation of how objective-reflective-normative thinking could emerge from the kind of thinking that humans share with non-human animals.

## Overview of *A Natural History of Human Thinking*

Tomasello calls the thinking that we share with non-human animals “individual intentionality” (2014: 4). Individual intentionality is what an animal exhibits if it cognitively represents experiences to itself ‘off-line’, simulates or makes inferences involving these representations, and self-monitors and assesses how these simulated experiences might lead to specific results so as to make an instrumentally rational decision on what to do to satisfy its own desires (Tomasello 2014: 9).

Tomasello mentions a number of studies that show that, e.g. great apes display individual intentionality. They are able to use cognitive representations of their physical surrounding for causal inferences, represent another agent’s intentional states, and employ the representation to make inferences pertaining to how the individual will act given the mental state she is in. Great apes also monitor their own cognition: based on their certainty about what they know, they assess their chances of success at a task and make decisions accordingly. Great ape thinking, which Tomasello takes to correspond to the thinking in our last non-human ancestors from 5 to 6 million years ago, is hence already relatively sophisticated.

It is, however, only geared toward the satisfaction of the animal’s own individualistic needs when it is competing with group mates for valued resources, Tomasello writes. He holds that great apes’ individual intentionality is only self-focussed “cognition for competition” of typically lone-acting creatures (2014: 31).

According to the “shared intentionality hypothesis” that Tomasello sets out to defend in *A Natural History of Human Thinking*, uniquely human thinking evolved from this self-focussed, individual intentionality as an adaptation for “dealing with problems of social coordination, specifically, problems presented by individuals’ attempts to collaborate and communicate with others” (2014: 4). He write that this evolution happened in two steps, one leading from individual to “joint intentionality” and the other from joint intentionality to “collective intentionality”, both of which are for Tomasello instances of human-unique “shared intentionality” (2014: 5–6).

The first evolutionary step occurred about 400,000 years ago, in early humans (the *Homo heidelbergensis*). Tomasello write that while humans’ great ape ancestors lived, just as contemporary great apes, mostly individualistic and competitive lives in which individual intentionality served them just fine, early humans could no longer survive without collaborating with each other in dyadic units when out foraging. The result was a species-unique selection for and evolution of skills and motivations to engage in cooperative activities, which relied on a “dual-level structure” consisting of “joint goals” i.e. goals that both interactants shared and knew they shared with each other—and “joint attention” i.e. both interactants were attending to the same thing and knew they both did—forming a “joint intentionality” of the moment (Tomasello 2014: 33, 38).

Since the different individuals involved in cooperative activities with this structure still retained different perspectives and had to play different roles for both to achieve success in joint tasks, the need for early humans to coordinate their actions and attention referentially on external situations and entities arose. Tomasello argues that this initiated the evolution of new forms of communication such as pointing, pantomiming, and iconic gestures via which interactants now started to inform the other of aspects of the environment relevant for her/him to achieve the joint goal.

These new forms of communication and collaboration in turn led to new forms of thinking. For instance, in early humans’ cooperative communication, both the communicator of a message and the recipient had to “anticipate”, Tomasello writes, the “perspective of their partner, which required socially recursive inferences that embedded the intentional states of one partner within those of the other” (2014: 72). Individuals had to “think about their communicative partner thinking about their thinking” because the communicator had to determine how best to convey to the recipient her intention, and the recipient had to reconstruct the communicator’s intention by appealing to what she wanted him to know, Tomasello maintains (2014: 59).

Furthermore, early humans’ collaborative activities involved partner choice. This meant that each individual developed an interest in being viewed as a good collaborator, for bad collaborators weren’t chosen as partners in foraging activities and hence ultimately faced starvation. Tomasello holds that each individual thus began to monitor and control her own acting and thinking with the other’s perspectives and evaluations in mind.

Still, early humans’ thinking was socially normative only in the sense that they were concerned with how their particular collaborative partner, rather than the group as a whole, assessed their cooperation and understood their communicative acts. Early humans didn’t yet subject themselves to any ‘objective’ normative standard of the group as a whole. Their thinking was thus “perspectival-recursive-socially monitored thinking”, but not yet objective-reflective-normative thinking (Tomasello 2014: 79). For the latter to enter the scene, second-personal, joint intentionality had to become “collective intentionality”, Tomasello writes (ibid).

In his account of the transition, the social groups that early humans formed were only loose pools of individuals for *ad hoc* dyadic collaborations. Two demographic factors changed this. First, competition with other human groups emerged. In order to protect their way of life from invaders, the unsteady social pools of early humans were thus forced to become uniform collaborator groups with the shared goal of group survival. Second, when human populations grew, smaller groupings that were still part of a culture separated from the rest. As a result, members of a particular group now encountered the problem of identifying individuals belonging to them.

Tomasello holds that in response to these two problems, modern humans started developing a group identity, demarcating the ‘we’ from the ‘them’, the competitor groups (2014: 82f). In order to enable the recognition of and coordination with in-group strangers with whom one had no personal common ground, local practices were conventionalised and became to function as shibboleths via which members of the group could be easily identified. Conventionalised practices as well as social norms and institutions to which each group member conformed and expected all others to conform then constituted a cultural common ground that provided the basis for collaboration with in-group strangers.

To further strengthen conformity and facilitate collaborations within the group, early humans’ iconic gestures became substituted with linguistic conventions, which, unlike early humans’ gestures, supported arbitrary connections between signs and referents allowing for abstract conceptualisations, Tomasello writes. Since the linguistic conventions were passed on to the next generation, the children of the group didn’t have to reinvent conceptualisations but inherited from their social environment various different ways of classifying the world for themselves and others. They learned to view the same situation and entity simultaneously under different guises, e.g. as an antelope by the tree, as an animal by the tree, as food by the tree, etc. This knowledge, accumulated over time in the social environment via reliable teaching and learning mechanisms, introduced *inter alia* the possibility for formal inferences as opposed to merely causal ones, for subjects could now think that given that there is, say, an antelope by the tree, there is an animal (or food) by the tree.

In addition, to be a good partner in collaborations, cooperative argumentation, and shared decision-making, which was vital for survival, individuals now also often had to make explicit in language their own attitudes toward particular contents (e.g. whether they were certain or doubtful about a proposition) and the reasons for their claims. To ensure the intelligibility and rationality of those linguistic acts and reasons, modern humans needed to simulate in advance the cultural group’s normative judgments of the intelligibility and rationality of the communicative acts and reasons in order to align them with the group’s standards.

In their self-reflection and self-monitoring, humans now referred to the normative perspective of all users of the linguistic conventions. For each of them took it that to be a member of the group, one must behave as the group as a whole does, i.e. follow the norms to which all are committed, or else be ostracised. Modern humans thus referred in their thinking and action planning to the “agent-neutral”, “‘objective’ perspective engendered” by their “cultural world” that then “justified personal judgments of true and false, right and wrong” (2014: 115). The collaboration and communication in modern humans were hence characterised by *collective* rather than merely second-personal, joint intentionality. They led to the evolution of reflective, ‘objective’, and normative, i.e. uniquely human thinking, Tomasello writes.

He ends the main discussion in his book by emphasising that skills of shared intentionality, e.g. the ability to engage in joint attention and form joint goals, are not innate but biological adaptations that come into being during ontogeny as the individual uses them to collaborate and communicate with others. This means that without social interactions during childhood, and without collectively created and transmitted cultural environments, including adults and all their cultural equipment (e.g. language), joint and collective intentionality won’t develop. As a result, uniquely human thinking won’t emerge either, Tomasello concludes.

## Critical discussion

The central argument of Tomasello’s book that uniquely human thinking evolved from individual intentionality in two steps that crucially involved a human-unique form of cooperation is illuminating and plausible. Overall *A Natural History of Human Thinking* is clearly written and a rewarding reading for anyone interested in the evolution of human cognition. The book fills an important gap in the literature by offering an empirically and theoretically well-supported account of the evolution of a very specific aspect of human cognition, i.e. objective-reflective-normative thinking, and its link to uniquely human cooperation.

While I’m largely sympathetic to Tomasello’s shared intentionality proposal, I have reservations about some of the details. I will focus only on his story about the evolution of one particular component of “objective-reflective-normative thinking”, namely subjects’ “socially recursive and self-reflective inferences about others’ or their own intentional states” (2014: 4). I will ignore the objective and normative aspects that Tomasello takes to be part of uniquely human thinking.

### Explicit versus implicit thinking

It is a common view that reading other people’s minds and engaging in socially recursive thinking evolved in competitive social worlds for Machiavellian purposes, i.e. for the purpose of or for guarding against deception and manipulation (Byrne and Whiten 1988, 1997). Tomasello argues for a different proposal. He claims that socially recursive thinking evolved because it was required for cooperative communication.

One kind of study that he mentions to support this involves the object-choice task (see Behne et al. 2005, 2012). For instance, if in interacting with a chimpanzee, the experimenter points to food on the ground, the chimp will follow the pointing and take the food. If the food is hidden in one of two buckets, however, and the experimenter then points to either of them, the chimp will look to the bucket but won’t make the inference that this is where the food is. Chimpanzees hence fail in the object-choice task to find the food. In contrast, in the same kind of setting, already 12-months old human infants immediately comprehend the informative motive underlying the adult’s pointing, and know that the pointed-to bucket is the one containing the sought-after object (Tomasello 2014: 52).

How are they able to know this? Tomasello argues that in the successful performance “in the object choice task […] the recipient [of the message] infers that the communicator *intends* that she *know* that the food is in the bucket—a socially recursive inference that great apes apparently do not make”^1^ (2014: 57). The inference at issue, Tomasello writes, “requires in all cases an abductive leap” such as: “his pointing in the direction of that otherwise boring bucket would make sense (i.e. would be consistent with common ground, relevance, and newness) if it is the case that he *intends* that I *know* where the reward is” (ibid).^2^ This abductive socially recursive inference enables already human infants to comprehend the experimenter’s pointing in the object-choice task and find the sought-after object, Tomasello claims. He holds that in the task, in “human cooperative communication” in general, individuals “must” engage in such inferences (2014: 59).

Suppose that early humans were indeed, as Tomasello suggests, forced to cooperatively communicate. If subjects need to engage in socially recursive thinking in order to cooperatively communicate (e.g. in the object-choice task), then Tomasello’s proposal that such thinking evolved in early humans for enabling cooperative communication seems plausible.

There is, however, reason to be sceptical about the claim that socially recursive thinking is required for this purpose. For instance, Tomasello holds that in the object-choice task, in order to grasp the communicated message, the recipient needs to infer that the communicator intends that she know that the sought-after object is in the bucket. Since the recipient of the message in the developmental psychology study that Tomasello cites is a 12-months-old infant (2014: 52), in his view, a 12-months-old infers that the adult pointing her to the bucket “*intends* that she *know*” that the sought-after object is in the bucket (2014: 57).

This proposal lacks psychological plausibility, however. An understanding of the intention that S knows that *p* requires the possession of some concept of knowledge because the propositional content of the intention explicitly refers to knowledge. Yet, there is no evidence that children acquire the concept of knowledge before the concept of belief (Butterfill 2013), which is thought to happen at around 3–4 years of age (Wellman et al. 2001).

Recent studies involving the violation-of-expectation paradigm and gaze tracking do indicate that infants as young as 7–15 months are able to register other subjects’ false beliefs (Onishi and Baillargeon 2005; Surian et al. 2007; Kovács et al. 2010).

But, on the basis of further experimental results, it is widely accepted that this early understanding of mental states is at best *implicit*, i.e. automatic and unconscious in nature (see, e.g. Low and Perner 2012; Schneider et al. 2015). No one so far claims that these infants form *explicit* representations of other’s mental states, i.e. representations that figure in subject-controlled and conscious processing (Pacherie 2013).

Since that is so, it is fair to say that the 12-months-olds in the object-choice task also don’t engage in explicit socially recursive thinking. If they don’t do so, however, then, against Tomasello’s claim, such thinking isn’t required for cooperative communication. For, as he grants, these infants do engage in cooperative communication in, e.g. the object-choice task.

Indeed, suppose that the child involved in the task makes the default assumption that in general an adult subject S will help her achieve her goals. When she is searching for the hidden object, and sees S point to one of the buckets, she will then infer from S’s behaviour that the object she is currently looking for is in the pointed-to bucket. To draw this inference, the child might simply treat S as a mindless machine that has the function to assist her in her projects and point her to the location of objects that she is looking for. That is, the child doesn’t need to represent, explicitly or implicitly, any mental states, let alone engage in socially recursive thinking in order to find what she is looking for. Similarly, if S makes the default assumption that making eye contact with the child and then pointing to an object will help her find the object, then S will be able to successfully communicate to her where the object is without any kind of meta-representational processing. Neither the infant nor S needs to engage in such processing to cooperatively communicate.

Furthermore, even if subjects had to start, e.g. implicit socially recursive thinking in order to cooperatively communicate, this still wouldn’t help Tomasello with his project in *A Natural History of Human Thinking*, for the socially recursive thinking whose evolutionary origin he wishes to explain requires *explicit* representations of mental states. It requires explicit representations because, as mentioned above, for Tomasello, instances of “thinking”, including socially recursive thinking, are “system 2 processes” (2014 4). And system 2 processes are in Kahneman’s (2011) dual-system account, which Tomasello endorses (2014: 4), explicit, subject-controlled and conscious in nature.^3^

Finally, since Tomasello’s avowed focus is on system 2, i.e. explicit thinking, his proposal that socially recursive thinking evolved because it is required for cooperative communication becomes also questionable from a phenomenological point of view. For it is often noted in the literature on social cognition that if the inferences involved in producing mental state attributions were “explicit, they should show up in our experience”, but “they rarely do” (Gallagher and Hutto 2008: 18). Typically, in social interactions, including cooperative communication, we aren’t aware of any mental states or inferences about what others or we intend or think. Whatever meta-representational processing might be involved, it clearly doesn’t need to be conscious but normally remains unconscious (Apperly 2010).

There is no reason to believe that things were any different in early humans. That is, early humans too will presumably have been able to engage in cooperative communication without explicit, conscious meta-representational processing. But if that is so, then Tomasello’s claim that “human cooperative communication is evolutionarily new” in that individuals “*must* think […] about their communicative partner thinking […] about their thinking [emphasis added]” (2014: 59), where thinking is understood as a “system 2 process” (2014: 4), is false.^4^

### Socially recursive inferences and egocentric biases

There is another reason for being sceptical about Tomasello’s proposal even if we ignore the distinction between implicit and explicit thinking. It relates to a particular kind of bias in communication. I will say a bit more about the bias first before returning to Tomasello’s view.

A number of studies show that in communication interactants tend to exhibit an “egocentric bias”: they have the tendency to take their own perspective to be automatically shared by the other (see, e.g. Nickerson 1999; Royzman et al. 2003; Epley et al. 2004; Keysar 2007; Birch and Bloom 2007; Lin et al. 2010; Apperly et al. 2010). Interestingly, this effect is particularly pronounced in interactions with close others.

For example, Savitsky et al. (2011) investigated whether listeners are more egocentric in communication with a friend than a stranger. They used a task in which a ‘director’ gives an addressee instruction to move items in an array, some of which are only seen by the addressee but not by the director. So, for instance, the director might tell the addressee to ‘move the mouse’—referring to a mutually visible computer mouse –, and to comply, the addressee then has to exclude a toy mouse that she can see but that she knows that the director can’t see. Savitsky et al. found that subjects who were given directions by a friend made more egocentric mistakes, i.e. they looked at and reached for an object only they could see, than those who followed directions provided by a stranger.

Similarly, in a second study, subjects who tried to convey particular “meanings with ambiguous phrases overestimated their success more when communicating with a friend or spouse than with strangers” (Savitsky et al. 2011: 69). These results suggest that subjects engage in “active monitoring of strangers’ divergent perspectives because they know they must, but […] they ‘let down their guard’ and rely more on their own perspective when they communicate with a friend” (ibid).

These findings^5^ challenge Tomasello’s proposal. On his view, there was a trend toward and selection of perspective taking and socially recursive thinking when early humans became interdependent, cooperative, and lived in “small-scale” groups in which each one knew the other (2014: 82f). Yet, the data suggest that perspective taking and socially recursive thinking in fact *decrease* in interactions with cooperative people with whom one is familiar and interdependent, e.g. spouses and friends, rather than strangers. In these situations, subjects seem to take their own perspective to be automatically shared by the other, and there is a trend *away* from perspective taking.

*Prima facie*, this is puzzling, for an egocentric bias threatens cooperative communication and increases the potential for miscommunication. Why do subjects nonetheless exhibit such a bias especially when interacting with close others?

The following proposal seems plausible. When interactants share the same environment and jointly attend to the same thing, what is accessible and salient to the communicator will usually be equally accessible and salient to the recipient. As a result, in these situations, an egocentric approach will support successful communication without requiring communicators and recipients to model each other’s perspective or mental states (Pickering and Garrod 2004; Barr and Keysar 2005; Lin et al. 2010). Recipients of a message can then anchor interpretation in their own perspective, and, if need be (e.g. in the case of a misunderstanding), employ information about the communicator’s perspective to incrementally adjust away from the anchor (Nickerson 1999; Epley and Gilovich 2001; Epley et al. 2004; Tamir and Mitchell 2013).

Does the recipient’s subsequent adjustment to the perspective of the communicator rely on representing his perspective?

It is well known that simultaneously forming and entertaining distinct mental models is difficult (see, e.g. Johnson-Laird 1983; Pickering and Garrod 2004). Perhaps a more realistic proposal is thus that in cooperative communication, subjects “externalise” computations about each other’s perspective and thinking (Pickering and Garrod 2004: 12, 21). That is, even though communicator and recipient *could* directly compute each other’s perspective, in cooperative groups, they both will receive plenty of feedback from each other on their performance. This will allow them to update their semantic representations on the basis of individual successes or failures to convey and comprehend messages without having to compute each other’s perspectives and knowledge states themselves. Social feedback mechanisms thus allow the interactants to ‘off-load’ cognitive work, i.e. computations pertaining to each other’s perspective, onto their social environment (Young 1998; Barr 2004).

There is evidence that such an externalisation of computations does indeed occur. Studies show, for instance, that listeners often ask speakers to clarify the reference of a term despite the fact that if they adopted the speaker’s perspective, they would find that their mutual knowledge uniquely defines the referent (Keysar et al. 2000; Keysar 2007). That is, “even when addressees are presented with clear cues to what is mutually known, they often opt to resolve ambiguity by engaging in an epistemic exchange [e.g. asking clarification questions and providing feedback] rather than computing the referent themselves” (Barr and Keysar 2005: 33).

Note that once the referent has been fixed interactively, and a precedent has been set, the subsequent use and comprehension of the communicative act won’t require mutual perspective taking or socially recursive thinking either. For interactants may then on each occasion refer back to the precedent.

Empirical work supports this view. Studies show, for instance, that listeners tend to interpret a referential expression according to naming precedents set by a previous speaker even when they are aware that the current speaker was not in fact present at the time when the precedents were established (Barr and Keysar 2002; Malt and Sloman 2004). In these cases, with anyone who was, just as the listener, present when the precedent was set, the listener will subsequently be able to successfully cooperatively communicate about the referent at issue without socially recursive thinking and perspective taking. The data hence speak against Tomasello’s view that in cooperative communication subjects “must” adopt the other’s perspective (2014: 59).

More generally, given the way Tomasello characterises early humans’ social life, one would expect that particularly the kind of early humans that he envisages externalised computations about each other’s mental states and exploited the feedback mechanism involved in their interactions. For, as noted, he holds that early humans lived in “small” groups and were “interdependent with one another in an especially urgent way” (2014: 137). Further, early humans were cooperative, assumed that the other too “had cooperative motives”, and were “each trying to help the other” to achieve the “joint goal of recipient comprehension” (Tomasello 2014: 73).

Now, in social interactions in which participants are interdependent, mutually assume that the other is cooperative, and mutually make an effort to ensure communicative success, communicators will evidently refrain from ambiguous and deceptive communicative acts. Furthermore, they will aim to make information transmission as efficient as possible, because this will, given their interdependence, benefit both interactants. Since perspective taking and thinking about thinking are computationally complex and cognitively effortful processes for both parties (Apperly et al. 2006; Epley and Caruso 2009; Lin et al. 2010), and since in cooperative communication interactive feedback tends to lead to effectively the same result without requiring the computational complexity and effort (Young 1998; Pickering and Garrod 2004; Barr 2004), one would expect that the early humans that Tomasello has in mind relied on each other’s feedback rather than socially recursive inferences in order to settle the meaning of communicative acts and ensure communicative success.

Unlike Tomasello’s view, this proposal manages to accommodate the data on a stronger egocentrism in cooperative communication with close others. For, assuming that Tomasello is right about his characterisation of early humans’ social environments, then due to the interdependence of early humans and the small size of the groups in which they lived, early human communicators and recipients will have received copious feedback from each other on their performance. These aspects of early humans’ social environments will have allowed early humans to be more egocentric and assume by default that close others share their own perspective. Since an egocentric bias will for them also have made their cognitive processing in cooperative communication with close others computationally more economical and tractable, it seems likely that this is why the bias evolved and is still present in contemporary humans.

In sum, then, the preceding points suggest that cooperative communication doesn’t necessarily require simulating what the other is thinking about one’s own thinking. They cast doubts on Tomasello’s proposal that socially recursive thinking evolved in groups of highly interdependent and cooperative individuals for *enabling* cooperative communication. It is more probable that the early humans that he considers evolved the disposition to anchor their interpretation of each other’s communicative acts onto their own egocentric perspective^6^ and then, in the case of a misunderstanding, adjusted away from it, off-loading meta-representational processing pertaining to each other’s perspective onto their social interactions. Since early humans arguably did not need to simulate the other’s thinking about their own thinking to cooperatively communicate, and since there is empirical evidence that cooperative communication can proceed without perspective taking (Barr and Keysar 2002; Malt and Sloman 2004), Tomasello’s proposal about the evolution of socially recursive thinking can be rejected.^7^

But why then did socially recursive thinking evolve? While this isn’t the place for a detailed answer, the early development of meta-representational capacities in infants, who aren’t typically confronted with uncooperative interactants, suggests that these capacities, including socially recursive thinking, evolved not so much for enabling cooperative communication, as Tomasello suggest, but rather for allowing infants to deal with another pressing problem they face, namely social learning.^8^

Social learning frequently requires that the learner “understand that a performance is stylised, that a crucial step has been slowed down, exaggerated, or repeated to make it more overt” (Sterelny 2012: 146). To ensure reliable knowledge transmission and acquisition, both the learner and the teacher “need to read each other” in that each “monitors the other and their joint focus of attention and intention” (ibid). That is, both need to engage in mutual perspective taking and socially recursive thinking. Given the important role of social learning in human infants, there is good reason to assume that socially recursive thinking evolved as an adaptation for it.

## Conclusion

Tomasello’s new book *A Natural History of Human Thinking* makes a plausible case for the view that the apparent uniqueness of our thinking is ultimately grounded in our species-specific dispositions and abilities to engage in collaboration and cooperative communication with each other. His overall argument would have benefitted if attention had been paid to the distinction between explicit and implicit thinking, and if the data on egocentric biases in communication had been considered. Having said that, Tomasello’s ideas on what makes human thought unique and what explains its origin are intriguing and likely to shape future debates on theses issues.^9^

## Footnotes

1 The italics in the quotes of this paper are original.

2 The reasoning from a general principle to what another subject intends that Tomasello here takes to be “required in all cases” in which subjects work out what someone else wants them to know seems to suggest that he advocates a theory–theory view of social cognition. In other places in the book, however, his use of the term ‘simulation’ suggests he favors a simulationist account (see also Tomasello 1999: 70), or a theory-theory/simulationist hybrid. In the following discussion, not much hinges on whether he endorses a theory-theory, simulationist, or hybrid view.

3 This point also holds with respect to the following proposal that Tomasello mentioned in personal communication. He writes: in their cooperative communication human infants are not tracking others beliefs (or knowledge based on beliefs) but merely what others have perceived and so are familiar with. The studies by Moll and Liebal that I cite show infants’ amazing ability to keep track of what they have experienced with specific other individuals and what they have not, and it is this—functionally equivalent to [the] implicit [mental state] understanding [infants exhibit in violation-of expectation tasks]—that infants are using at the early stages. If infants’ ability to keep track of what others have experienced is *implicit* or ‘functionally equivalent’ to implicit processing about mental states, then the data Tomasello mentions yield little support for claims about *S2*, i.e., *explicit*, conscious meta-representational thinking and its evolution. That is, the data lend little support to his project. If, alternatively, one wishes to claim that this ability involves in fact explicit meta-representational processing, then further arguments or, better, evidence are needed than those that Tomasello provides in his book.

4 In his discussion of collective intentionality, Tomasello offers a second proposal on why conscious meta-representational thinking evolved. He holds that in discourse, to be a good collaborator, one often needs to provide others with an insight into one’s own propositional attitudes toward the contents that one communicates. Tomasello suggests that this requires making one’s attitudes explicit in language, which in turn only works if one can consciously think about them first (2014: 102f, 117, 139). However, there is reason to doubt Tomasello’s proposal, for one can often convey one’s mental states to others by *expressing* (rather than reporting) them, which doesn’t require meta-representations of them to be conscious, see Rosenthal (2012).

5 There is more evidence for the point that egocentrism is stronger in interactions with close others, leading *inter alia* to a felt transparency of one’s own mind to them; see, e.g., Vorauer and Cameron (2002), and Cameron and Vorauer (2008).

6 Goldman (2006) holds that when S exhibits an egocentric bias, this is the result of a “quarantine failure”: in the simulation process, the subject fails to isolate her own perspective from that of the other, and so the former seeps into the latter (165). That is, on his view, when S is in communication egocentrically biased, then she still engages in perspective taking or simulation. However, note that even Goldman acknowledges that such a case is a “limiting case” of simulation in which “the simulation element is null” (41). Given this, there is no reason to accept that simulation takes place at all, rather than a direct attribution, see also Wallin (2011).

7 It is worth noting that there are various ways in which cooperative communication might *appear* to rely on perspective taking even though no perspective-taking abilities but other processes are involved, see, e.g., Barr (2014) for an interesting discussion and a list of “impostors” of perspective taking.

8 Tomasello (1999) himself proposes that socially recursive thinking evolved for social learning. Curiously, in *A Natural History of Human Thinking*, he doesn’t consider the view.

9 I would like to thank Nick Shea, Kim Sterelny, and Michael Tomasello for very helpful comments and clarifications on a previous draft of the paper.
